# Supplementary material for: Distinct Changes in Gut Microbiota Are Associated with Estradiol-Mediated Protection from Diet-Induced Obesity in Female Mice
Source: Metabolites. 2021 Jul 30;11(8):499. doi: 10.3390/metabo11080499 (PMC8398128; doi:10.3390/metabo11080499)
Supplement: Supplementary file 1 [file metabolites-11-00499-s001.zip › metabolites-1276011-supplementary.pdf]

Supplementary Materials

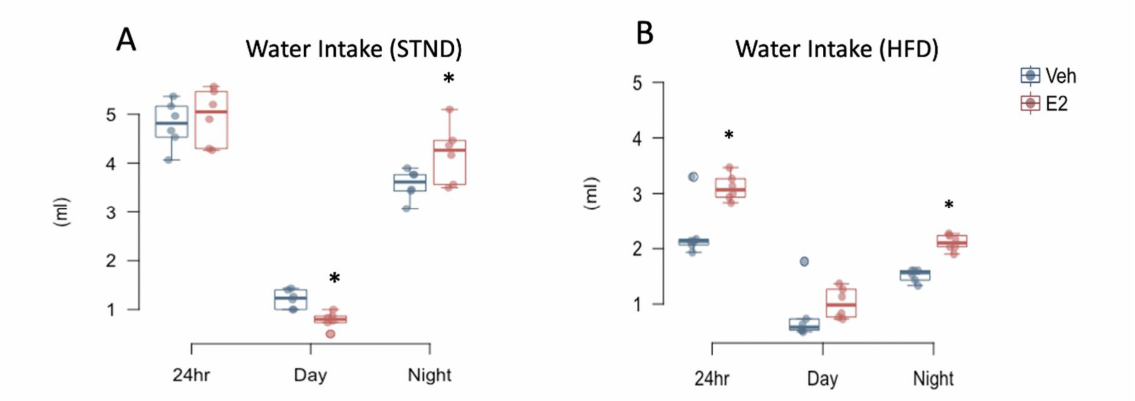

Figure S1. Estradiol alters water intake in female mice during STND (A) and HFD (B). Mice were kept in metabolic cages from days 11–13 after ovariectomy and E2 implant and the average 24-h data were obtained from 72-h data and used for statistical analysis. * indicates differences between E2 and Veh mice (n = 6/group) ($p < 0.05$, *t*-test).

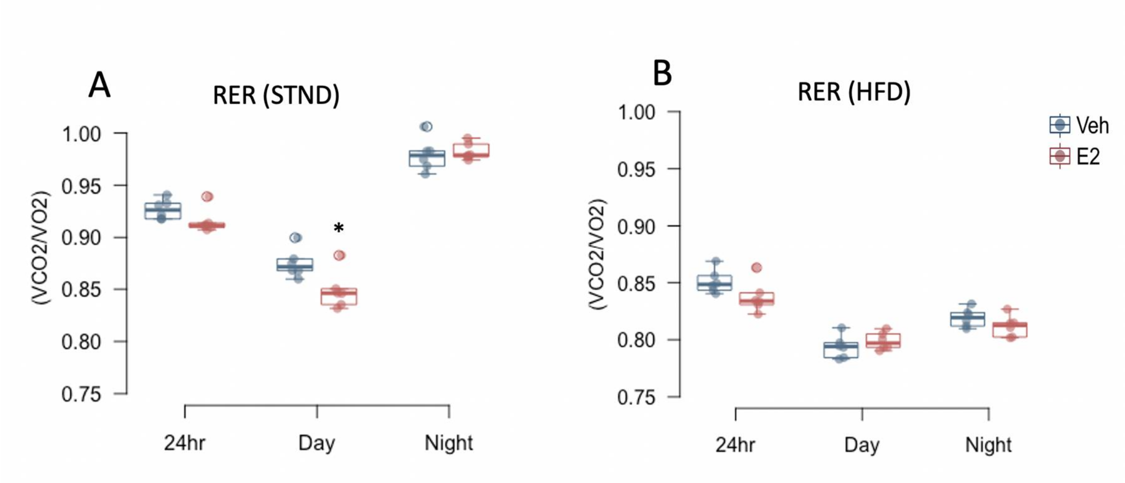

Figure S2. Estradiol decreases the respiratory exchange ratio (RER) in female mice during the day. Respiratory exchange ratios of mice on STND (A) or HFD (B) were measured in metabolic cages for 72 hrs. The average 24-h data were obtained from 72-h data and used for statistical analysis. * indicates differences between E2 and Veh mice (n = 6/group) ($p < 0.05$, *t*-test).

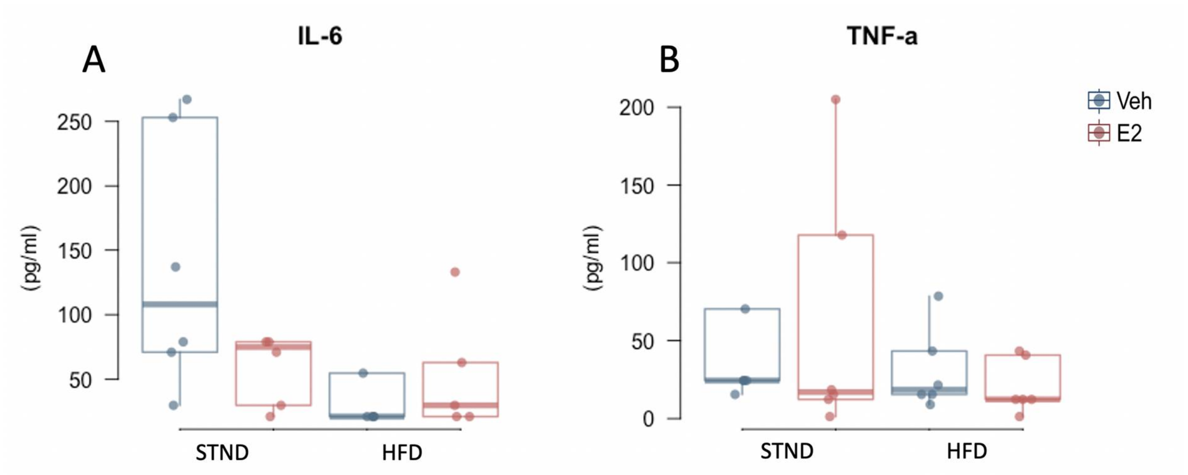

Figure S3: Estradiol or high fat diet did not alter levels of the plasma cytokines, IL-6 and TNF-α in female mice. 5 h-fasting blood samples were used to measure IL-6 (A) and TNF-α (B) during STND (on D8) and HFD (on D23); n = 6/group.

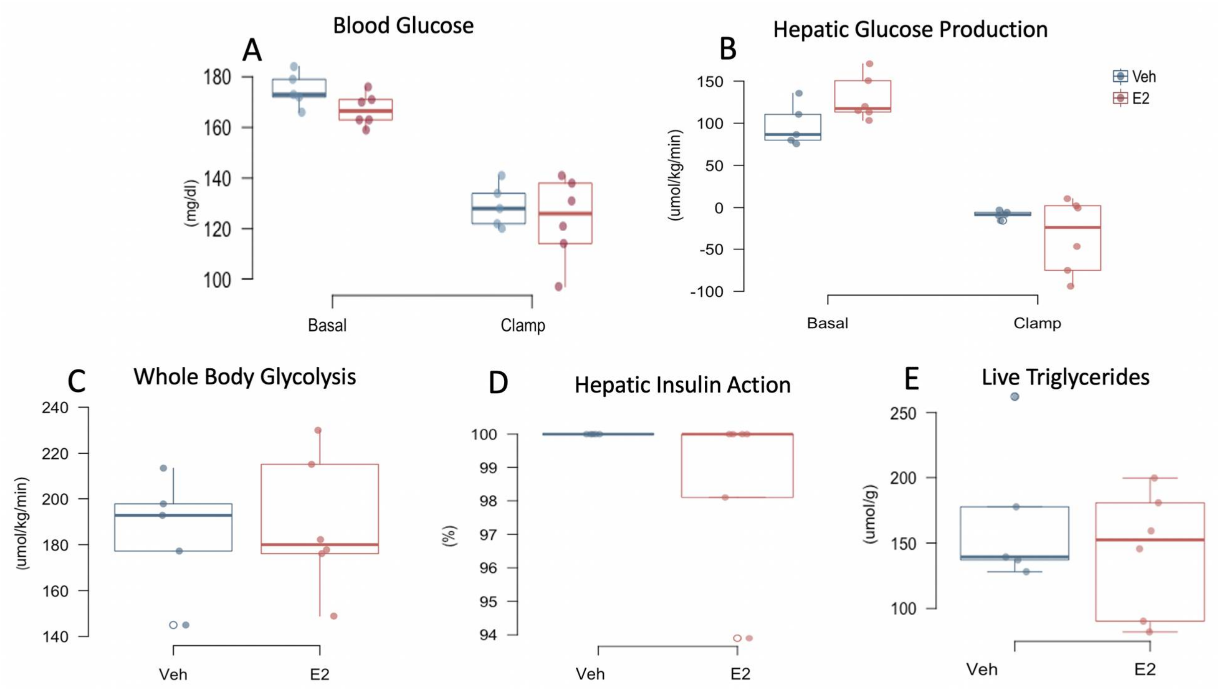

Figure S4. Estradiol does not alter hepatic insulin sensitivity and lipid production in female mice on HFD. Mice E2 (n = 6) and Veh (n = 5) underwent hyperinsulinemic-euglycemic clamp on days 43–45, a week following jugular vein surgery. Blood glucose (A) Whole-body glycolysis (B) Hepatic glucose production (C) Hepatic insulin action (D) Liver triglycerides (E).

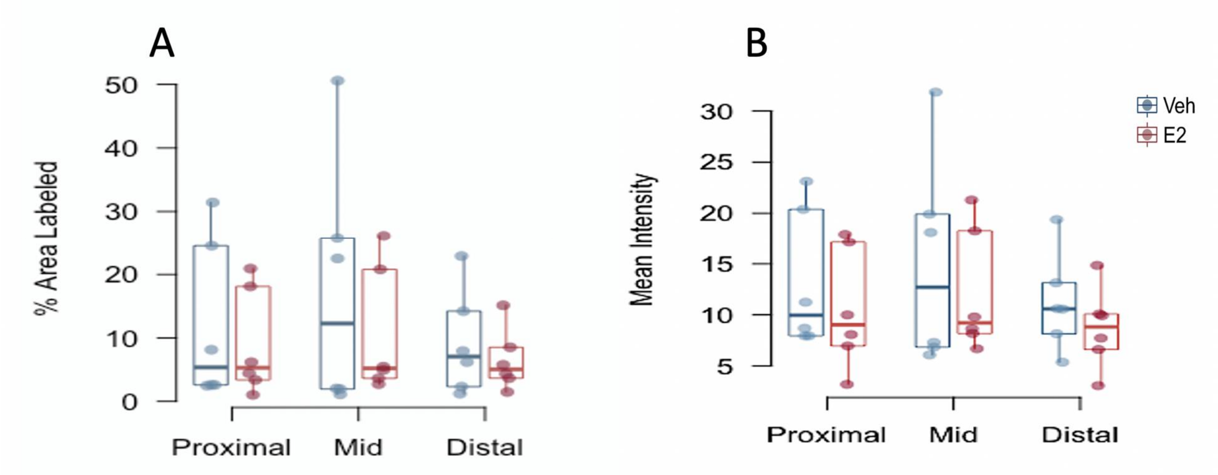

Figure S5. Estradiol does not alter zona occludens (ZO-1) immunoreactivity in the colonic epithelium in female mice fed a HFD. Percent area (A) and mean intensity (B) in the three subdivisions of the colon (n = 6/group).

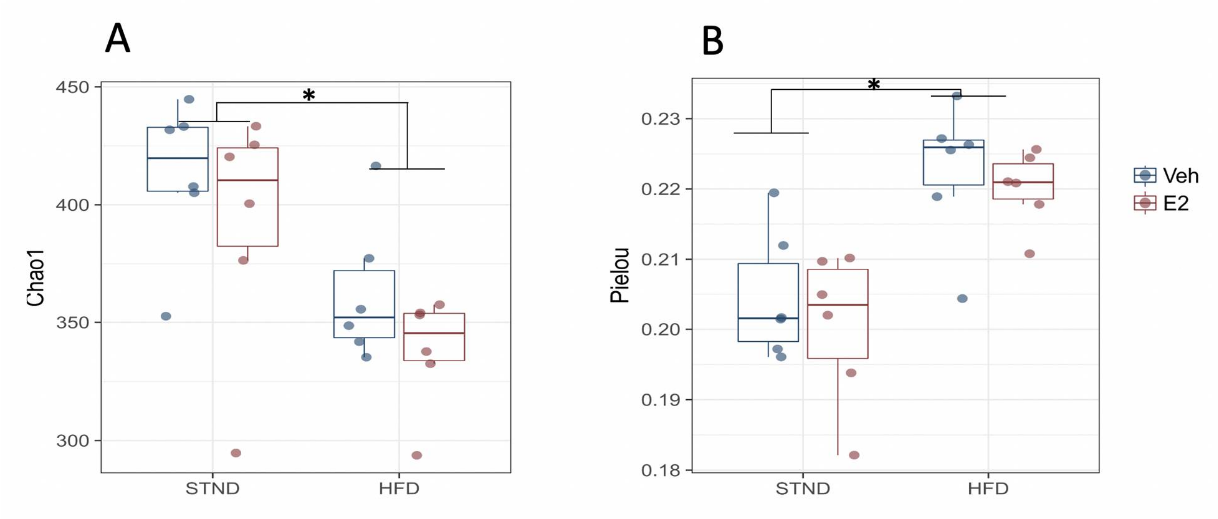

Figure S6. HFD alters gut microbiota α-diversity in female mice. HFD lowers richness (A) and increases evenness (B), (n = 12/group). * indicates a difference between STND vs. HFD ($p < 0.05$; “lme” in regression).
